# Osteoporosis and dry eye syndrome: A previously unappreciated association that may alert active prevention of fall

**DOI:** 10.1371/journal.pone.0207008

**Published:** 2018-11-05

**Authors:** Yu-Ting Jeng, Shu-Yi Lin, Hsiao-Yun Hu, Oscar K. Lee, Li-Lin Kuo

**Affiliations:** 1 Department of Ophthalmology, Taipei City Hospital, Taipei, Taiwan; 2 Department of Education and Research, Taipei City Hospital, Taipei, Taiwan; 3 Institute of Public Health and Department of Public Health, National Yang-Ming University, Taipei, Taiwan; 4 Institute of Clinical Medicine, National Yang-Ming University, Taipei, Taiwan; 5 Zhongxiao Branch, Taipei City Hospital, Taipei, Taiwan; 6 Department of Health Care Management, National Taipei University of Nursing and Health Sciences, Taipei, Taiwan; Fondazione Toscana Gabriele Monasterio, ITALY

## Abstract

**Objective:**

Osteoporosis is a multifactorial disease associated with inflammation and hormone imbalance. It is noteworthy that dry eye syndrome shares a similar pathophysiology with osteoporosis. Both diseases are more prevalent among the elderly and females. Dry eye syndrome can result in impaired vision, which increases the risk of fall and fracture when osteoporosis exists. In this study, we investigated whether osteoporosis is associated with an increased risk of developing dry eye syndrome.

**Methods:**

Claims data from the National Health Insurance Research Database (NHIRD) of Taiwan were used to conduct a retrospective population-based cohort study covering the period from January 1, 2000, to December 31, 2011. Multiple logistic regression was used to determine whether osteoporosis is an independent factor in the risk of developing dry eye syndrome, with risk estimates presented in the form of odds ratios (ORs).

**Results:**

The exclusion of patients with specific autoimmune diseases and those younger than 50 years old resulted in 42,365 patients in the osteoporosis group and 147,460 patients in the comparison group during the study period. The number of patients newly diagnosed with dry eye syndrome was 6,478 (15.29%) in the osteoporosis group and 15,396 (10.44%) in the comparison group. The crude OR of patients with osteoporosis developing dry eye syndrome was 1.55 and the 95% confidence interval (95% CI) was 1.50–1.60. After adjusting for patients’ age, sex, and underlying comorbidities, the adjusted OR was 1.26 and the 95% CI was 1.22–1.30. Subgroup analysis revealed this association in each age group and among females but not among males.

**Conclusions:**

Our results demonstrate that osteoporosis is a risk factor for the subsequent development of dry eye syndrome. Clinicians should be aware of the early symptoms of dry eye syndrome in osteoporotic patients in order to prevent further complications.

## Introduction

Dry eye syndrome refers to aqueous underproduction or over-evaporation decreasing the lubrication of ocular surfaces, which can result in superficial punctate keratopathy, corneal filaments, conjunctival scarring, or even corneal melting. Patients with dry eye syndrome experience ocular discomfort, visual disturbance, and pain. This has been shown to reduce the quality of life, impair physical functioning, and retard work performance [1]. The reported prevalence of dry eye syndrome varies from 4.87% to 33.7%, depending on which population being studied and which study being cited [2, 3]. In the United States, the cost of dry eye syndrome on society has been estimated at US$55.4 billion [4]. Multiple factors have been implicated in dry eye syndrome, including ocular inflammation, systemic disease, medical or surgical history, medication, lifestyle, and environment [5]. The sex hormones, androgen and estrogen, have both been shown to affect lacrimal gland and meibomian gland function in the regulation of tear film [6, 7].

Osteoporosis is a major health issue characterized by decreased bone mass, disruption of bone microarchitecture, and increased bone fragility, all of which increase the risk of fracture. The lifelong incidence of fracture caused by osteoporosis is approximate 40–50% in women and 13–22% in men [8]. In patients aged 65 or older suffering from hip fracture, the mortality rate is 3-fold higher than in the general population [9]. The economic costs of osteoporotic fracture include surgical fees, hospitalization, rehabilitation, and indirect costs like work productivity [10]. Osteoporosis is also considered a multifactorial disease, associated with genetic factors, age, sex, systemic disease, and diet [11].

Preventing fractures in patients with osteoporosis is a key objective of care. Visual disturbance is one complication of dry eye syndrome shown to hamper the safety of patients in their daily activities, including the risk of fall [12, 13]. It is noteworthy that osteoporosis and dry eye syndrome share common risk factors and epidemiological characteristics. The prevalence of both disorders is higher among females and has been shown to increase with age [14–17]. Inflammation and degeneration have been shown to underlie both diseases; however, few studies have mentioned a correlation between the two diseases.

Our objective in this study was to determine whether patients with osteoporosis are at greater risk of developing dry eye syndrome, and to alert active prevention of unfavorable complications in the elderly, using a nationwide population-based database.

## Materials and methods

### Database and population

In 1995, the Taiwanese government launched a social health system referred to as National Health Insurance (NHI), which serves as the single payer of medical expenses in Taiwan. Enrollment in NHI is compulsory and currently includes 23 million people, which represents approximately 99% of the national population. Construction of the National Health Insurance Research Database (NHIRD) takes into account the privacy of patients and data security. In this study, we employed the Longitudinal Health Insurance Database (LHID) 2000, which comprises all claims data from 1 million NHI beneficiaries selected at random in 2000. Sample selection was overseen by the National Health Research Institute (NHRI) with the aim of ensuring that there would be no significant differences between the sample and the overall population of NHI beneficiaries in terms of various factors, such as gender and age. Claims data are available from 1995 and all enrollment files are de-identified. This large nationwide cohort provides an excellent study resource. In this work, the LHID 2000 dataset was used as the basis to examine the period from January 1, 2000, to December 31, 2011. The coding of diseases was conducted in accordance with the International Classification of Diseases, Ninth Revision, Clinical Modification (ICD-9-CM), based on ambulatory care and inpatient discharge records. This study was approved by the NHRI and the Research Ethics Committee (REC) of Taipei City Hospital (case number: TCHIRB-10701114-W). The REC waived the requirement of written consent.

### Study group and outcomes

**Fig 1:**
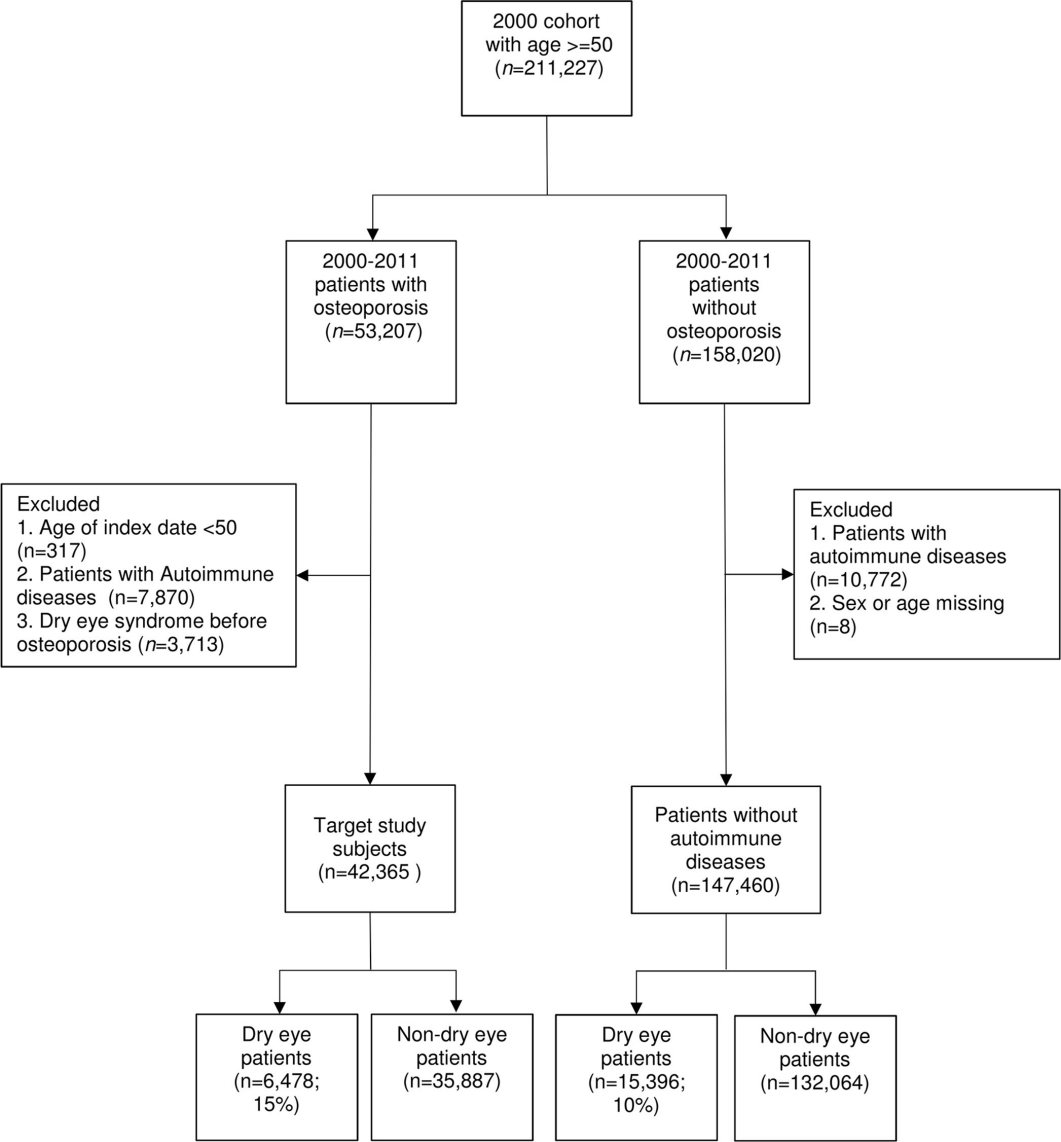
Flow chart of patient selection.

Patients who were 50 years or older during the study period were included in the cohort. All patients diagnosed with osteoporosis (ICD-9-CM: 733.X) after 2000 were enrolled as the study group, and the remainder of the population was assigned to the comparison group. Patients with missing data were excluded, as were patients with autoimmune diseases, including those affecting connective tissue (ICD-9-CM: 710.X), namely systemic lupus erythematosus or Sjögren's syndrome, rheumatoid arthritis (ICD-9-CM: 714.X), Wegener's granulomatosis (ICD-9-CM: 446.4), cystic fibrosis (ICD-9-CM: 277.X), Marfan syndrome (ICD-9-CM: 759.82), and osteogenesis imperfecta (ICD-9-CM: 756.51). The osteoporosis and comparison groups were followed up until the occurrence of dry eye syndrome (ICD-9-CM: 375.15) or the end of the study period. A detailed flow chart of the study is presented in Fig 1.

Pre-existing comorbidities previously associated with dry eye syndrome were categorized as secondary dry eye, including conjunctival scarring (ICD-9-CM: 372.6X), ocular pemphigoid (ICD-9-CM: 694.61), Steven Johnson syndrome (ICD-9-CM: 695.1X), and post-corneal transplant (ICD-9-CM: V42.5). In addition, pre-existing comorbidities which were prone to develop osteoporosis were gathered, including vitamin D deficiency (ICD-9-CM: 268.9), Cushing’s syndrome (ICD-9-CM: 255.X), hyperthyroidism (ICD-9-CM: 252), and hyperparathyroidism (ICD-9-CM: 242.X).

### Statistical analysis

Descriptive analysis was used to compare the osteoporosis group and comparison group in terms of demographic data and the incidence of dry eye syndrome during the study period. The risk of developing dry eye syndrome was calculated as an odds ratio (OR) using logistic regression. Multiple logistic regression was used to adjust for age, sex, and comorbidities. Crude OR and adjusted OR were both estimated. We also conducted analysis of OR in terms of demographic data and comorbidities. In all of the results, statistical significance was defined as a *P* value of less than 0.05. Data analysis was conducted using SAS for Windows version 9.4 (SAS Institute, Cary, North Carolina, USA).

## Results

The flow chart of patient selection was demonstrated in Fig 1. A total of 211,227 patients aged 50 years or older were included in this study, including 53,207 patients with diagnosis of osteoporosis and 158,020 patients without it. After excluding patients with autoimmune disease, those presenting dry eye syndrome prior to the diagnosis of osteoporosis, and patients with missing data, a total of 42,365 patients were included in the osteoporosis group and 147,460 patients were included in the comparison group.

Demographic data and clinical characteristics are listed in Table 1. The majority of the osteoporosis group was female (32,216 of 42,365 patients, 76.04%) but a minority in the comparison group (57,607 of 147,460 patients, 39.07%, *P*<0.0001). The mean age was 65.66 ± 9.96 years in the osteoporosis group and 63.10 ± 10.40 years in the comparison group (*P*<0.0001). The prevalence rates of comorbidities of interest were all higher in osteoporosis group than in comparison group. In osteoporosis group, there were 265 patients had secondary dry eye (0.63%, *P*<0.0001), 7 patients had vitamin D deficiency (0.02%, *P* = 0.0639), 1,115 patients had had Cushing’s syndrome (2.63%, *P*<0.0001), 119 patients had hyperparathyroidism (0.28%, *P* = 0.0116), and 2,453 patients had hyperthyroidism (5.79%, *P*<0.0001).

**Table 1:** Demographic data and clinical characteristics (n = 189,825).

Variable	Total	Osteoporosis (n = 42,365)	Non-osteoporosis (n = 147,460)	P value
		n (%)	n (%)	
Gender				<0.0001
Male	100,002	10,149 (23.96%)	89,853 (60.93%)	
Female	89,823	32,216 (76.04%)	57,607 (39.07%)	
Age (mean±SD)		65.66±9.96	63.10±10.40	<0.0001
50–59	78,280	13,282 (31.35%)	64,998 (44.08%)	
60–69	55,720	13,760 (32.48%)	41,960 (28.46%)	
70–79	40,016	11,266 (26.59%)	28,750 (19.50%)	
≧80	15,809	4,057 (9.58%)	11,752 (7.97%)	
Comorbidity				
Secondary dry eye	933	265 (0.63%)	668 (0.45%)	<0.0001
Vitamin D deficiency	16	7 (0.02%)	9 (0.01%)	0.0639
Cushing’s syndrome	3,115	1,115 (2.63%)	2,000 (1.36%)	<0.0001
Hyperparathyroidism	427	117 (0.28%)	310 (0.21%)	0.0116
Hyperthyroidism	6,798	2,453 (5.79%)	4,345 (2.95%)	<0.0001

During the study period, 21,874 patients were newly diagnosed with dry eye syndrome, including 6,478 (15.29%) in the osteoporosis group and 15,396 (10.44%) in the comparison group, as shown in Table 2. The crude OR for patients with osteoporosis to develop dry eye syndrome was 1.55 (95% confidence interval, 95% CI, 1.50–1.60, *P*<0.0001). After adjustment for patient age, sex, and underlying comorbidities, the adjusted OR was 1.26 (95% CI, 1.22–1.30, *P*<0.0001).

**Table 2:** Incidence of dry eye syndrome in osteoporosis and comparison cohort (n = 189,825).

	Development of dry eye					
	Yes	No	Crude		Adjusted^¶^	
	n (%)	n (%)	OR (95%CI)	P value	OR (95%CI)	P value
Comparison	15,396 (10.44%)	132,064 (89.56%)	1.00		1.00	
Osteoporosis	6,478 (15.29%)	35,887 (84.71%)	1.55 (1.50–1.60)	<0.0001	1.26 (1.22–1.30)	<0.0001

^¶^Adjusted for gender, sex, and comorbidities of interest

The subgroup study, stratified by demographic data and comorbidities, was done using multiple logistic regression, as shown in Table 3. The adjusted OR for patients with osteoporosis to develop dry eye syndrome was significantly higher in the female group (adjusted OR, 1.33, 95% CI, 1.28–1.38, *P*<0.0001) and in all age-specific subgroups (age 50–59, 1.34; age 60–69, 1.25; age 70–79, 1.12; age 80 or more, 1.34). However, no significant difference was found in the male group (adjusted OR, 1.03, 95% CI, 0.95–1.11, *P* = 0.4781). No significant difference was found in all comorbidity groups either.

**Table 3:** Adjusted odds ratio of incidence of dry eye syndrome stratified by demographic data and comorbidities.

Variable	n	Adjusted^¶^ OR	95%CI	P value
Gender				
Male	100,002	1.03	(0.95–1.11)	0.4781
Female	89,823	1.33	(1.28–1.38)	<0.0001
Age				
50–59	78,280	1.34	(1.27–1.41)	<0.0001
60–69	55,720	1.25	(1.18–1.33)	<0.0001
70–79	40,016	1.12	(1.04–1.20)	0.0021
≧80	15,809	1.34	(1.13–1.58)	0.0006
Comorbidity				
Secondary dry eye	933	0.98	(0.68–1.40)	0.8945
Vitamin D deficiency	16	3.15	(<0.01->99.99)	0.9937
Cushing’s syndrome	3,115	1.21	(0.96–1.53)	0.1041
Hyperparathyroidism	427	0.84	(0.47–1.53)	0.5761
Hyperthyroidism	6,798	1.10	(0.97–1.25)	0.1226

^¶^ The multiple logistic regression model was adjusted for gender, age, secondary dry eye, vitamin D deficiency, Cushing’s syndrome, hyperparathyroidism, and hyperthyroidism

## Discussion

In this nationwide population-based cohort study, we identified an increased risk of developing dry eye syndrome among patients with osteoporosis, even after adjusting for comorbidities (OR = 1.26, 95% CI = 1.22–1.30, *P*<0.0001). Subgroup analysis revealed that this risk was found in female group (OR = 1.33, 95% CI = 1.28–1.38, *P*<0.0001) and in all age-specific groups.

The production of tears of a suitable composition is crucial for maintaining the stability of tear film. The three layers of tear film serve different functions, all of which are important to the health of ocular surfaces. The inner mucin layer protects the cornea and gives the hydrophobic corneal epithelium hydrophilic surface. The middle aqueous layer forms the bulk of tear film and contains immunoglobulins and nutrients. Finally, the outer lipid layer retards the evaporation of tears and decreases the surface tension of tear film [18]. Any form of dysfunction among these three layers can result in tear film instability. There is a long list of causes of dry eye syndrome, including ocular surface conditions (e.g., blepharitis, meibomian gland dysfunction), ocular surgical history (e.g., corneal transplantation, refractive surgery), inflammatory conditions (e.g., allergies), hormone imbalance (e.g., thyroid hormones, sex hormones), environment (e.g., dry weather, contact lens over-wearing, smoking), neurological conditions (e.g., trigeminal nerve palsy, infrequent blinking), medication (e.g., anti-histamines), and systemic disease (e.g., Sjogren's syndrome, diabetes mellitus) [19]. Dry eye syndrome is therefore considered a complex multi-factorial disease.

Our results are consistent with previous epidemiological studies, which have revealed that female patients and the elderly are prone to dry eye syndrome, an unsurprising observation given that organ function gradually declines with age [14, 17]. Aqueous deficiency caused by lacrimal gland dysfunction, tear over-evaporation caused by decreased lipid production, and decreased blink rate caused by decreased corneal sensitivity are all common pathophysiologies behind dry eye syndrome [20]. Chronic inflammation and oxidative stress of the lacrimal gland have also been observed. Among the elderly is a higher incidence of meibomian gland dropout and meibomian gland with atrophic morphology, which is regarded as a non-obstructive cause of decreased lipid production [21]. Anatomical changes of the eyelid, including eyelid retraction or eyelid malposition, can also lead to corneal exposure and dry eye syndrome.

The pathophysiologic relationship between osteoporosis and dry eye syndrome is complex; however, a number of mechanisms have been proposed. The first mechanism is a deficiency of sex hormones. Estrogen and androgen have both been shown to play important roles in the development of osteoporosis and dry eye syndrome [8, 22]. A drop in serum estrogen levels in menopausal women increases the number and activity of osteoclasts, which has an effect on the metabolism of bone [23]. Bone mineral density in patients presenting premature ovarian failure is lower than among women without this condition [24]. *In vitro* findings have revealed that androgen can stimulate the proliferation of osteoblast precursors [25]. Androgen deficiency and androgen deprivation therapy have also been shown to promote osteoporosis in men [26]. Conversely, estrogen and androgen replacement therapies have proven effective in the treatment of osteoporosis [27, 28]. The predominance of females in dry eye syndrome has been reported in many studies [5, 22]. Studies have also revealed an association between dry eye syndrome and both premature ovarian failure and androgen deficiency [6, 7]. It has been postulated that sex hormones regulate the meibomian gland and thereby influence the stability of tear film and indeed, the expression of androgen and estrogen receptors has been observed in the meibomian gland [29, 30]. Animal studies have revealed that androgen stimulates lipid productionhttps://www.ncbi.nlm.nih.gov/pubmed/16579987 whereas estrogen decreases lipid secretion in the meibomian gland [31, 32]. However, research into the effects of hormone replacement therapy on tear production has generated conflicting results. Affinito et al. and Scuderi et al. reported that estrogen hormone replacement therapy was beneficial to Schirmer test results; however, Erdem et al. failed to observe any benefits [33]. Topical or transdermal testosterone supplements have been shown to improve dry eye syndrome in some studies [34, 35]. Future randomized controlled trials will be needed to determine the effects of sex hormone supplement on dry eye syndrome; however, our findings suggest that targeting sex hormones may be an effective therapy for both diseases. We failed to observe a correlation between osteoporosis and dry eye syndrome among males in this study. Given the fact that meibomian gland dysfunction and evaporative dry eye often occur during menopause and aging, deficient tear secretion from both androgen deficiency and menopause-related estrogen deficiency may be an important etiologic factor in the pathogenesis of dry eye syndrome in women [5, 7].

Purinergic signaling is another possible mechanism underlying both diseases. Purinergic receptors receive stimulus from adenosine or adenosine triphosphate (ATP), and are respectively categorized as P1 and P2 receptors. P2 receptors can be further divided into P2X and P2Y receptors, based on their activity. The fact that nucleosides and nucleotides are intracellular molecules have led researchers to the conclusion that their existence in the extracellular space was associated with stress signals [36]. A number of studies have reported that purinergic signaling can affect dry eye syndrome as well as osteoporosis. Purinergic receptors can be found in the ocular surface. Under stimulus from P2Y2 receptors, the tear film tends to be more stable due to an increase in the production of mucin [37–40]. Thus, inflammation-related ocular disorders can be corrected through the restoration of tear film [41]. From the perspective of osteoporosis, activation of P2Y2 receptors has been shown to inhibit osteoblast activity and retard bone formation [42]. Mice lacking the P2Y2 receptor present elevated bone mineral content [43]. Wesselius contradicted previous indications that P2Y2 receptor can have harmful effects on bone mass. He reported a non-significant decrease in bone mineral density associated with one type of single nucleotide polymorphism associated with the P2Y2 receptor gene [44].https://www.ncbi.nlm.nih.gov/pmc/articles/PMC3568433/ Clopidogrel, a P2Y12 antagonist, was also shown to increase the prevalence of osteoporotic fractures in a cohort study [45]. One *in vitro* study also reported that Clopidogrel treatment led to a reduction in osteoblast as well as osteoclast function [46].

Various elements in the aqueous layer of the tear film, including proteins, vitamins, and electrolytes, contribute to a healthy ocular surface [47]http://onlinelibrary.wiley.com/doi/10.1111/j.1444-0938.2011.00634.x/pdf. Calcium in the tear film are particularly important in cell signaling, metabolism, gene expression, osmolarity, and even the function of conjunctival goblet cells [48, 49]. Evidence suggests that calcium ointment reduces the symptoms of dry eye [50]. Nonetheless, hypocalcemia in patients with osteoporosis indicates the possibility of underlying hypothyroidism, vitamin D deficiency, or poor gastrointestinal absorption. This is a clear indication that maintaining adequate calcium level is crucial to treating both of these diseases.

Researchers have shown an association between vitamin D deficiency in the elderly and osteoporosis as well as dry eye syndrome [51, 52]. Vitamin D is crucial to the regulation of calcium homeostasis, bone metabolism, immune modulation, inflammation control, cell proliferation, and cell differentiation [53, 54]. Vitamin D has also been linked to enhanced production of anti-inflammatory cytokines, such as IL-10 and IL-13, as well as reductions in the production of pro-inflammatory cytokines, such as TNF-α and IL-8 [55, 56]. Immune cells, such as T-cells, dendritic cells, and monocytes, are also regulated by vitamin D [57]. Thus, vitamin D may inhibit the underlying inflammatory cascade of osteoporosis and dry eye syndrome. Studies have shown that vitamin D supplements can be helpful in the treatment of both diseases [51, 58]. In Taiwan, vitamin D is classified as dietary supplement and is not covered by the NHI. Because of this reason, vitamin D deficiency was not properly shown in the claims data, as coding of diagnosis is related to reimbursement. This is indeed the limitation of retrospective claims data. In our future prospective study, serum 25(OH)D levels will be measured and their correlation with osteoporosis and dry eye will be evaluated. Nonetheless, when we take into account the issue of inflammation, it would be reasonable to expect that the link between the two diseases would be stronger in patients with vitamin D deficiency.

Osteoporosis and dry eye syndrome can both be divided into primary and secondary diseases based on the underlying pathology. Secondary osteoporosis is commonly linked to endocrine disorders, such as hyperparathyroidism and Cushing’s syndrome, whereas secondary dry eye syndrome is generally linked to ocular surface diseases, such as conjunctival scarring or post-ocular surgery [11, 19]. These secondary causes of both diseases were viewed as comorbidities of interest in our study. However, we had relatively few patients with comorbidities in this study, such that no significant difference was found regarding the adjusted OR for each comorbidity. Last but not least, our results demonstrate that this association occurred in all age groups, which is indicative of an age-independent correlation.

Falling in patients with osteoporosis is associated with consequent disabilities, while visual impairment is an important contributor to falling [10, 13]. Our study demonstrated that patients with osteoporosis are at higher risk of developing dry eye syndrome. This risk is observed in patients aged 50 years or older and female gender. It is important to raise awareness of this condition, especially in doctors treating osteoporosis.

Visual disturbance is one complication of dry eye syndrome shown to hamper the safety of patients in their daily activities, including the risk of fall [12, 13]. It is noteworthy that osteoporosis and dry eye syndrome share common risk factors and epidemiological characteristics. The prevalence of both disorders is higher among females and has been shown to increase with age [14–17]. Inflammation and degeneration have been shown to underlie both diseases; however, few studies have mentioned a correlation between the two diseases.

Previous literatures have indicated the relationship between dry eye syndrome and possible underlying autoimmune diseases, such as systemic lupus erythematosus, Sjögren's syndrome, rheumatoid arthritis, or Wegener's granulomatous [59, 60]. Renal tubular acidosis, a complication of Sjogren's syndrome, may cause low bone mineral density, predisposing these patients to osteoporosis [61]. For more precise investigation of the relationship between osteoporosis and dry eye syndrome, patients with confirmed autoimmune diseases were excluded in our study. The relationship between dry eye and osteoporosis in patients with autoimmune diseases are beyond the reach of our study.

This study had a number of limitations that must be taken into account. First, the diagnosis of osteoporosis and dry eye syndrome were determined by ICD coding obtained from NHI claims data. Because of the limitations of using administrative data, there were a limited number of coding columns. Thus, in cases of inaccurate disease coding and in cases where coding for the disease of interest was in latter columns, the status of the patient would be misinterpreted. Furthermore, the fact that the data were de-identified precludes data validation. Several disease parameters, such as the severity of the disease, were unavailable as was any indication of the treatment that patients actually received, thereby hampering any attempts at investigating the relationship between the severity of dry eye syndrome and osteoporosis. Second, our study included only patients aged 50 years or older; therefore, our results are not generalizable to younger patients. Third, the prevalence of comorbidities of interest was relatively low, which meant that any discussion of these comorbidities would be hampered by a lack of clinical data. Finally, our study population was made up entirely of Taiwanese. Thus, generalizability to other ethnic groups will require further study.

## Conclusions

Osteoporosis is associated with increased risk of developing dry eye syndrome, which can cause blurred vision and increase the risk of fall and fracture. The fact that both diseases are more prevalent in older populations means that any association between them could be of considerable importance to clinicians. We suggest that physicians treating patients with osteoporosis look for signs of dry eye syndrome early on. In cases of detection, patients should be referred to ophthalmologists to reduce the likelihood of blurred vision, falling, and unfavorable outcomes.
